# Redefining the catalytic HECT domain boundaries for the HECT E3 ubiquitin ligase family

**DOI:** 10.1042/BSR20221036

**Published:** 2022-10-04

**Authors:** Emma I. Kane, Steven A. Beasley, Johanna M. Schafer, Justine E. Bohl, Young Sun Lee, Kayla J. Rich, Elizabeth F. Bosia, Donald E. Spratt

**Affiliations:** Gustaf H. Carlson School of Chemistry and Biochemistry, Clark University, 950 Main Street, Worcester, MA 01610, U.S.A.

**Keywords:** HECT domain, HECT E3 ubiquitin ligase, multiple sequence alignment, ubiquitin, ubiquitylation, UniProt

## Abstract

There are 28 unique human members of the homologous to E6AP C-terminus (HECT) E3 ubiquitin ligase family. Each member of the HECT E3 ubiquitin ligases contains a conserved bilobal HECT domain of approximately 350 residues found near their C-termini that is responsible for their respective ubiquitylation activities. Recent studies have begun to elucidate specific roles that each HECT E3 ubiquitin ligase has in various cancers, age-induced neurodegeneration, and neurological disorders. New structural models have been recently released for some of the HECT E3 ubiquitin ligases, but many HECT domain structures have yet to be examined due to chronic insolubility and/or protein folding issues. Building on these recently published structural studies coupled with our in-house experiments discussed in the present study, we suggest that the addition of ∼50 conserved residues preceding the N-terminal to the current UniProt defined boundaries of the HECT domain are required for isolating soluble, stable, and active HECT domains. We show using *in silico* bioinformatic analyses coupled with secondary structural prediction software that this predicted N-terminal α-helix found in all 28 human HECT E3 ubiquitin ligases forms an obligate amphipathic α-helix that binds to a hydrophobic pocket found within the HECT N-terminal lobe. The present study brings forth the proposal to redefine the residue boundaries of the HECT domain to include this N-terminal extension that will likely be critical for future biochemical, structural, and therapeutic studies on the HECT E3 ubiquitin ligase family.

## Introduction

In 1995, a novel class of enzymes containing an approximate 350 residue conserved domain at their respective C-terminus were found to be homologous to the C-terminal domain of E6-associated protein (E6AP) and had the capabilities of forming a thioester bond with ubiquitin [1]. The most unique aspect of this study revealed that a highly conserved catalytic cysteine residue within this C-terminal domain was responsible for polyubiquitin chain formation. This discovery led to the classification of the homologous to E6AP C-terminus (HECT) E3 ubiquitin ligase family, with currently 28 human members that differ in size and the presence of various N-terminal protein–protein interaction domains involved in substrate recruitment [2]. Further investigation through structural analysis revealed this 350 residue domain was bilobal with numerous conserved residues within the hinge region found between the N*-* and C-terminal lobes that provide flexibility to support HECT-dependent ubiquitylation activity [3]. This cleft region is suggested to also be critical to its HECT function as loss-of-function mutations in E6AP within this region have been linked to Angelman syndrome [4]. Both lobes were also identified to have unique characteristics involved in ubiquitin handling by the HECT E3 ubiquitin ligases – where the N*-*terminal lobe is responsible for E2 ubiquitin conjugating enzyme recruitment and binding, while the C-terminal lobe contains the highly conserved catalytic cysteine residue that facilitated ubiquitin transfer onto a specific substrate [5,6]. Conservation within the hinge between the N- and C-terminal lobes have been suggested to allow for the flexible rotation to bring the catalytic cysteine residue in the HECT C-terminal lobe into close proximity with the E2–ubiquitin complex bound to the HECT N-terminal lobe [6]. Studies have also suggested a competing proximal addition model where the HECT domain can form higher oligomeric structures to facilitate polyubiquitin chain formation [7–11].

Members of the HECT E3 ubiquitin ligase family have been broadly categorized into three subfamilies based upon their domain architecture in their variable N-terminal protein–protein interaction domains and their functional similarities. These subfamilies include the neuronal precursor cell-expressed developmentally down-regulated 4 subfamily (NEDD4, 9 members), the HECT and RLD domain subfamily (HERC, 6 members), and the unclassified ‘other’ subfamily (13 members) [12]. The NEDD4 E3 ubiquitin ligases possess a Ca^2+^-binding C2 domain for phospholipid recognition [13] and two-to-four WW domains required for binding specifically to proline-rich motif-containing substrates [14]. Members of the HERC subfamily are divided based upon size, with only the large HERCs (HERC1/HERC2) containing more than one RCC1-like domain (RLD) for multiple substrate recognition and binding opportunities [15]. The remaining 13 HECT E3 ubiquitin ligases have diverse protein–protein interacting domains within their N-terminus, with some homology existing such as ankyrin repeat and armadillo repeat domains [2]. Thorough phylogenetic analyses of the HECT family suggest that it may be more appropriate to have 16 separate HECT subfamilies, including the notable division of small and large members of the HERC subfamily [16,17].

HECT E3 ubiquitin ligases are involved in a myriad of biological processes and cellular pathways including protein degradation, DNA damage repair, apoptosis, subcellular localization, and immunological response [2,18–20]. Numerous studies have implicated the dysfunction of members from the HECT E3 ubiquitin ligase family in disease development such as various cancers, immunological disorders, neurodevelopmental disorders, and neurodegenerative disease [18,21–24]. Since the HECT domain possesses the most prominent functional regions of this protein family, it is logical that a research focus would be directed towards structural and functional characterization of this catalytic region of the HECT E3 ubiquitin ligases. New structural insights into the HECT domain have shown that the introduction of extending the N-terminal sequence of the HECT domain is important for folding. While many of these crystal structures exist for HECT domains with an additional α-helix [5,25–27], the HUWE1 structure contains up to three α-helices [27].

Here, we show through bioinformatic analysis and biochemical characterization that up to four N-terminal α-helices beyond the ∼350 residue UniProt defined boundary are predicted to be present within all 28 HECT E3 ubiquitin ligases and that including these N-terminal extensions for the HECT domain improves solubility and increases ubiquitylation activity. Our bioinformatic evaluation of the various members of the HECT E3 ubiquitin ligases through multiple sequence alignment (MSA) with Jalview [28] coupled with secondary structure prediction and evaluating available PDB structures using PyMol [29] suggests there exists at least one amphipathic α-helix – with some predicted to have two to four α-helices – prior to the N-terminal lobe of the HECT domain. Our assertion is further supported by the recent crystal structures of the HECT domains of apoptosis-resistant E3 ubiquitin protein ligase-1 (AREL1, PDB: 6JX5) and ubiquitin-protein ligase E3C (UBE3C; PDB: 6K2C) HECT domains that included an additional 50 amino acids preceding the N-terminal lobe to the isolated HECT domains [25,30]. Our studies coupled with recent structural reports on different HECT domains indicate that the currently annotated UniProt HECT domain residue boundaries need to be revisited and revised.

## Results

**Figure 1:**
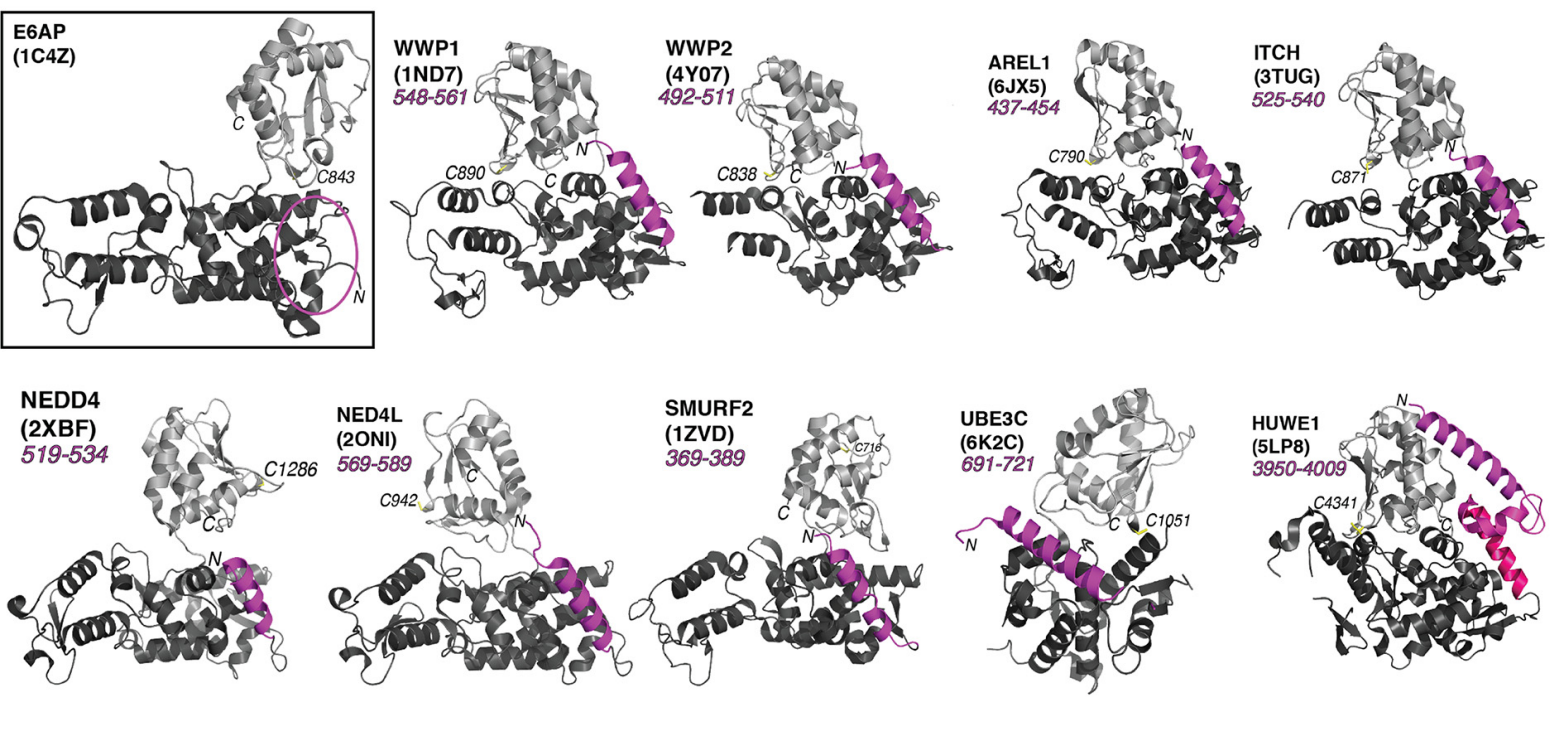
Structural analysis of the solved HECT domains models of various HECT E3 ubiquitin ligases Representative extended HECT domain models within the HECT E3 ubiquitin ligase family. With the amphipathic α-helix included, all models show a similar tertiary structure to its namesake E6AP (PDB: 1C4Z). The bilobal structure is shown with the C-lobe containing the catalytic cysteine (light gray), the N-lobe with the E2∼ubiquitin binding interface and sites required for oligomerization (dark gray), and the N-terminal α-helical extension (magenta). These crystal models are also missing some atoms including loops and the C-terminal tail of the HECT domain.

**Figure 2:**
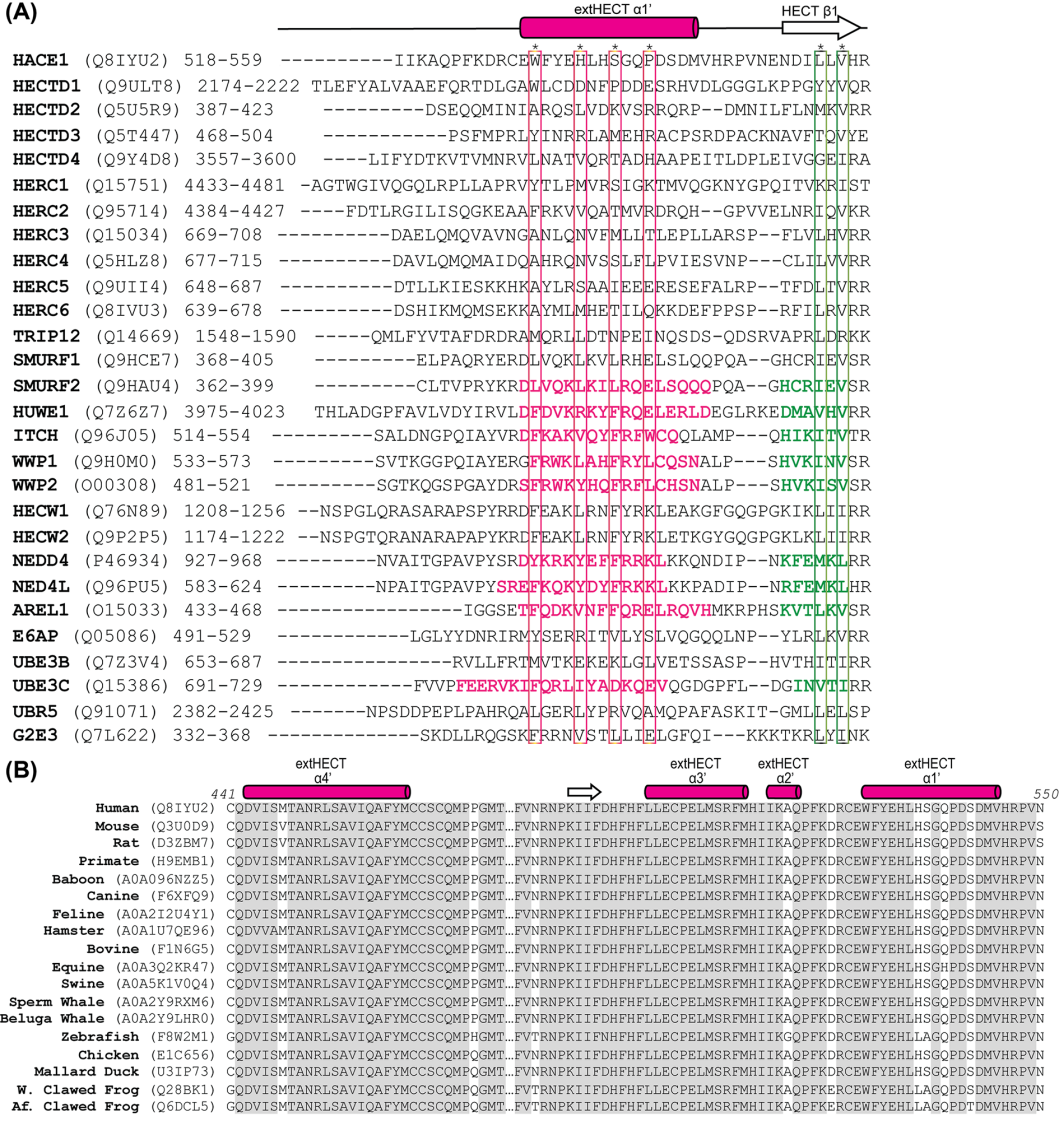
Paralog and ortholog MSA of the HECT E3 ligase family with secondary structure prediction through Jalview and JPred (**A**) Paralog MSA of the junction with the extended HECT α-helix (α1′) and the initial β-sheet (β1) of the HECT domain. Sequences and residue numbers were obtained through UniProt for all human HECT E3 ubiquitin ligases. Structural elements from solved crystal models with the α-helical extension (magenta) and N-lobe β-sheet (green) are indicated. Residues facing towards the hydrophobic core are designated with an asterisk. The alignment was produced using ClustalW and T-Coffee and manually curated by Jalview. (**B**) Ortholog results revealed high conservation for the HECT E3 ubiquitin ligase HACE1 where all four α-helices are predicted to fold preceding the N*-*lobe of the HECT domain. This finding compliments the paralog analysis shown in panel (A). Residues with absolute conservation are shown in gray.

In the years since the original E6AP HECT domain structure was published in 1999, every solved HECT structure has included at least one α-helical extension immediately upstream from the defined N-terminal lobe (Figure 1) [5,6,25–27,30–33]. The additional α-helix (often referred to in the literature as α1′) sits astride the HECT N-terminal lobe on the opposite face from the E2 enzyme binding site. The ClustalW and T-Coffee MSA of the 28 human HECT paralogs resulted in an alignment that includes a JPred predicted α-helix (Figure 2).

**Figure 3:**
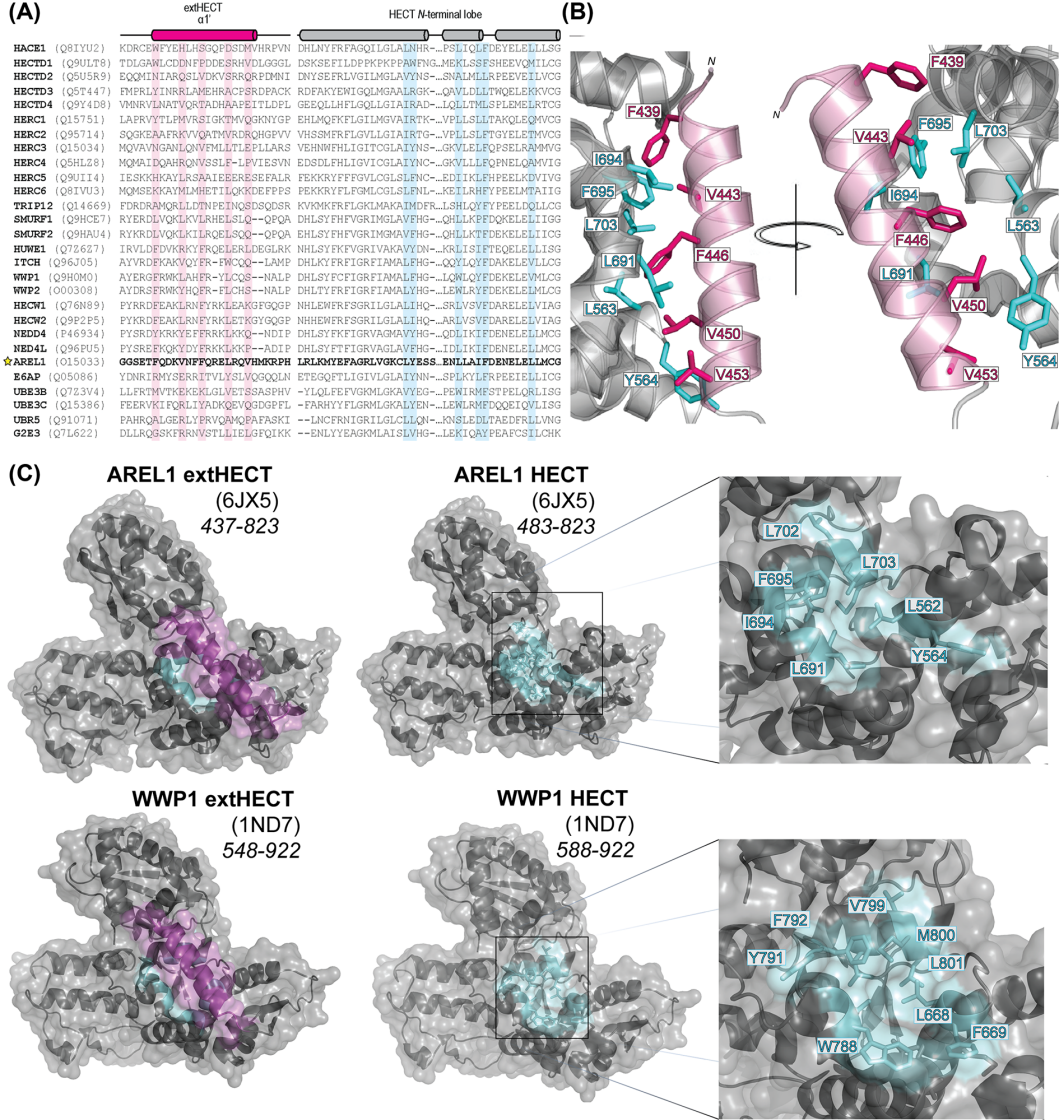
Secondary structure prediction reveals a hydrophobic pocket protected by the N-terminal α-helical extension (**A**) MSA analysis coupled with secondary structure prediction for all HECT E3 ubiquitin ligases indicates a conserved amphipathic α-helix (shown in magenta) with hydrophobic residues suggested to interact with residues found in the hydrophobic cleft of the HECT N*-*terminal lobe (shown in cyan). This alignment takes into consideration the conservation and secondary structure prediction with a focus on the 1′ α-helix preceding the HECT domain. (**B**) The AREL1 HECT (PDB: 6JX5) with conserved hydrophobic residues shown within the predicted α-helical extension (magenta) are predicted to interact with the conserved N-lobe residues (cyan). This alignment also confirms the role that extending the known HECT domain boundaries are important for domain stability and solubility. (**C**) Surface maps of the extended (right) and UniProt defined (left) WWP1 and AREL1 HECT domains. The conserved sidechain HECT N-lobe residues highlighted in panel (B) reside within the hydrophobic pocket (cyan). The α-helical extension (magenta) reduces the surface area exposed from the hydrophobic patch, as observed through the decrease in color. The inset (right) shows a closer look at the conserved hydrophobic residues in the hydrophobic patch.

The predicted α-helix shows a periodicity for the highly conserved hydrophobic residues resulting in their collective orientation on the same face of the α-helix. Further analysis of the HECT domains with known structures reveals these residues form a hydrophobic core along with the hydrophobic residues from three distinct α-helices found in the HECT N-terminal lobe that are also conserved (Figure 3A). For example, in the AREL1 HECT domain structure, these comprise F439, V443, F446, L450, and V453 on the α-helical extension interacting with residues L563, Y564, L691, L692, I694, F695, and L703 on the HECT N-terminal lobe creating a hydrophobic pocket (Figure 3B,C). Further structural analysis revealed that removing this protective helix exposes a contiguous hydrophobic patch for all the HECT proteins analyzed. For example, AREL1 has a hydrophobic patch measured to be 627 Å and WWP1 has a hydrophobic patch of 698 Å (Figure 3C). This exposed pocket may have contributed to the insolubility of the shorter constructs. It is interesting to note that the equivalent hydrophobic patch residues of the originally published E6AP structure missing the N-terminal α-helix (PDB: 1C4Z) are found in the core of the trimeric crystal interface.

**Figure 4:**
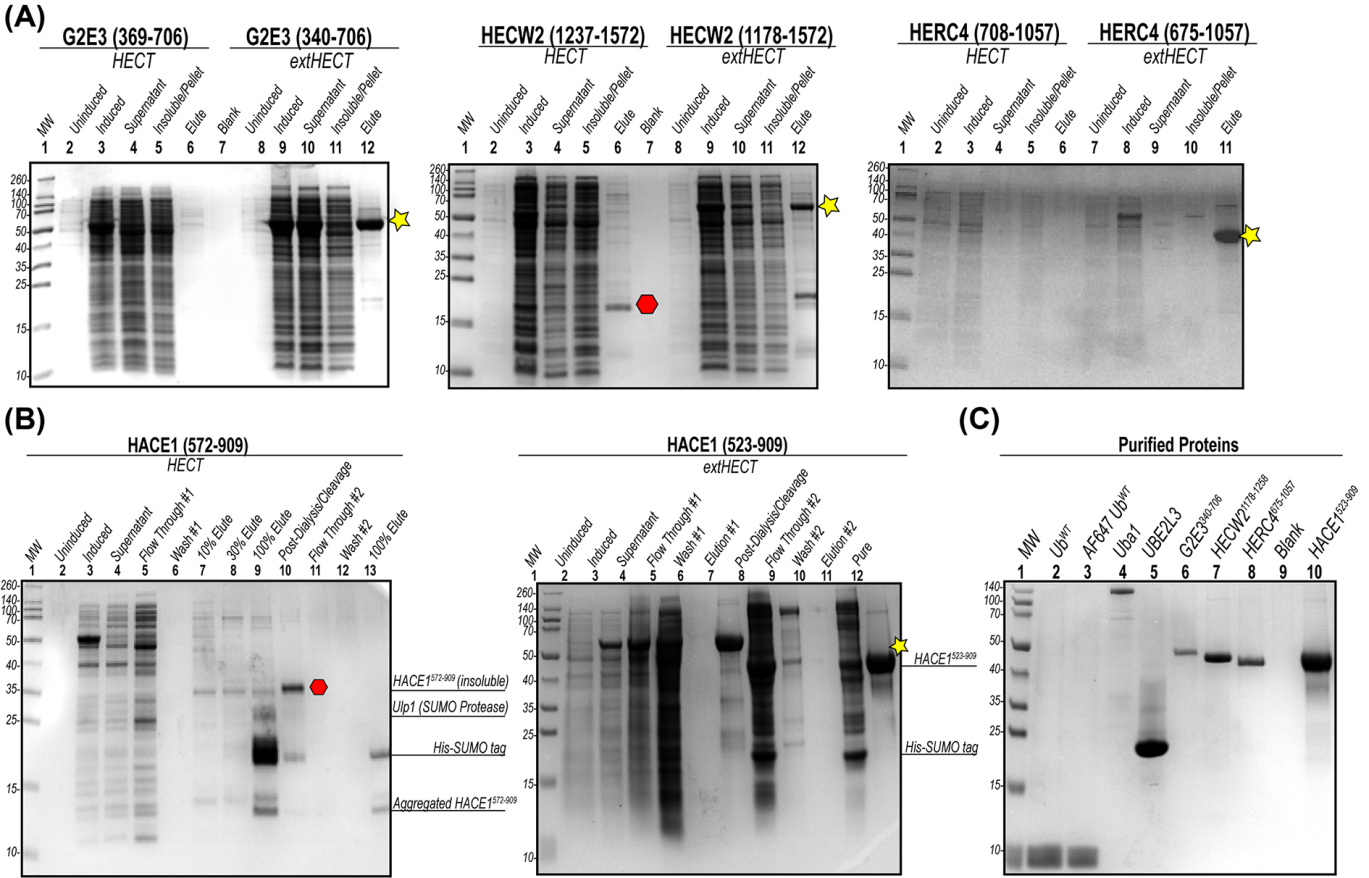
Solubility and purification of UniProt defined HECT domain against extended HECT domains (**A**) The solubility of each HECT and extHECT construct were analyzed by SDS-PAGE. Solubility following induction showed that the extHECT constructs of the HECT E3 ubiquitin ligases evaluated in this paper (shown with yellow stars) were more soluble than the shorter HECT domains using the domain boundaries annotated on UniProt. (**B**) Example SDS-PAGE of the purification steps for HACE1 HECT (residues 574–909 based on UniProt defined domain boundaries) and extHECT (residues 523–909) constructs. The HECT construct was deemed to be insoluble (shown with red octagon) while the extHECT HACE1 was significantly more soluble and with a higher yield (shown with yellow star). (**C**) Analysis of protein purity for all proteins used in this study including the isolated extHECT constructs. All samples were loaded at 5 mg of protein, except for UBE2L3 and HACE1^523-909^ which were loaded at 10 mg.

The protection of this hydrophobic pocket probably provides structural integrity to the HECT domain, which is supported by the marked increase in solubility we observed for our extended HECT domain constructs (Figure 4A). Each extension of the HECT domain we incorporated into our extended HECT (extHECT) expression constructs was based off the location of predicted boundaries of α-helices, with the inclusion of each α-helix showing improved solubility and HECT-dependent activity. Taken together, our MSA results support and further demonstrate that identical or similar residues within these corresponding positions within the predicted α-helix extension are also found in all 28 human members of the HECT E3 ubiquitin ligase family.

The HECT E3 ubiquitin ligases we assessed in this study (HECW2, HERC4, G2E3, and HACE1) showed significantly increased solubility upon induction with IPTG and higher yields when comparing our extHECT constructs to the current UniProt HECT domain boundaries (Figure 4). Our results demonstrate that the susceptibility of the exposed hydrophobic cleft at the N*-*terminus of the HECT domain can be mitigated by the addition of these additional α-helices. Each hydrophobic residue within the extended α-helix/helices suggests that this conserved region is required to support normal HECT-dependent ubiquitylation. The solubility of each HECT E3 ubiquitin ligase appears to be dependent on the addition of at least one of these predicted α-helical extensions. Both paralog and ortholog secondary structure prediction indicate that the extended region would likely have α-helical content (Figures 2 and 3).

The overexpression and purification of heterologous proteins in *Escherichia coli* greatly depends on the overall stability and native fold, while mitigating solvent accessibility of hydrophobic regions of the expressed protein [34–36]. Despite our repeated attempts to modify buffers, reagents, and purification procedures, we were unable to improve stability, activity, and/or the yield of any of our original HECT constructs using the UniProt defined domain boundaries. Our experimental optimization included (i) varying temperatures for expression following IPTG induction, (ii) altering IPTG concentrations, (iii) scaling down production culture volumes, and (iv) subcloning the HECT domain construct to be expressed with other purification fusion tags. While we were able to successfully purify some of these UniProt defined HECT constructs, the overall yield we obtained for our new extHECT constructs resulted in approximately a 50-fold increase in protein yield (Figure 4).

**Figure 5:**
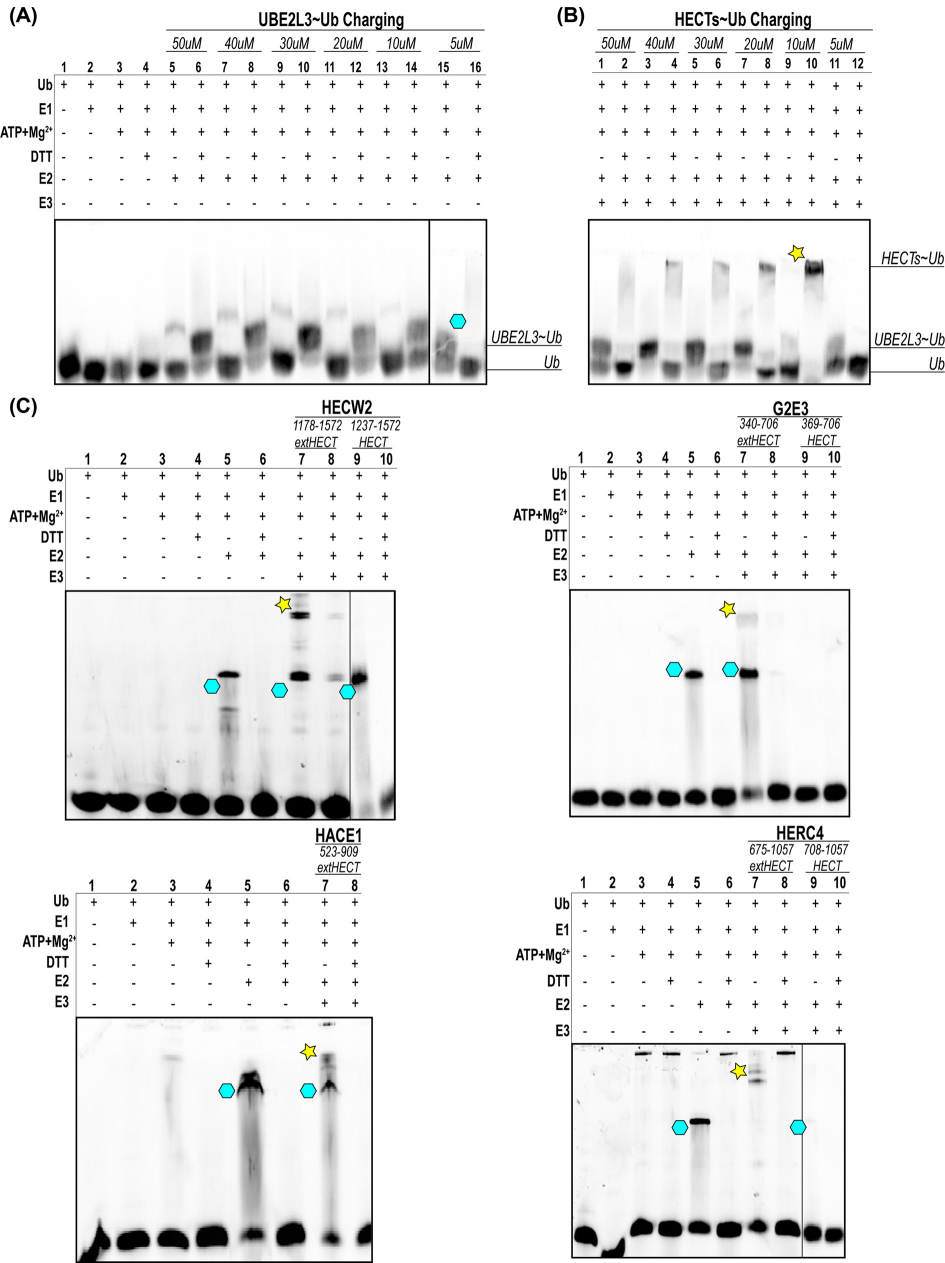
Ubiquitin activity assays show that the extHECT domains are more active than the UniProt defined HECT domain boundary constructs (**A**) Assay optimization through varying concentrations of Alexa Fluor 647-ubiquitin:UBE2L3 to assess the ideal concentration to minimize unused free ubiquitin. The UBE2L3∼ubiquitin complex (shown in cyan) is highlighted to demonstrate the ideal concentration of UBE2L3 used with minimal free ubiquitin present. (**B**) The UBE2L3:HECT/extHECT ratios were repeated as before to determine the ideal E3∼ubiquitin charging ratio (yellow star). (**C**) Fluorescent ubiquitylation activity assays of each HECT E3 ubiquitin ligase evaluated in this study. Gel images were taken with an iBright FL1000 gel imager with fluorescence settings for Alexa Fluor 647. The images were converted to black/white for ideal visualization of each ubiquitin transfer step and product.

We also assessed the ubiquitylation activity of our HECT and extHECT proteins using an *in vitro* fluorescent ubiquitylation assay that has been optimized to demonstrate E2∼ubiquitin charging/discharging and improved extHECT∼ubiquitin charging. We observed a marked increase in ubiquitylation activity by our extHECT constructs, whereas the HECT domains showed little to no activity (Figure 5). All extended HECT domains used for these assays demonstrated that the HECT domain could be charged with ubiquitin. To demonstrate optimization of UBE2L3∼Ub charging, UBE2L3 discharging and HECT/extHECT∼Ub charging, we performed an additional assessment to find the optimal concentration of UBE2L3 to ubiquitin to limit the excess free ubiquitin found at the bottom of the gel. Once determining the ideal concentration of ubiquitin (5 μM) and UBE2L3 (10 μM), we performed an optimization assessment of the E3 at a 1:1 ratio of UBE2L3 to reproducibly show E3∼ubiquitin charging and UBE2L3 discharge of ubiquitin. We found that using a HECT or extHECT concentration at 10 μM at a 1:1 ratio showed the robust activity of our extended HECT domain constructs. With the difficulty in isolating the shorter HECT proteins, our data shows that the inclusion of additional N-terminal α-helices provide overall greater structural integrity coupled with increased solubility that aid in HECT-dependent ubiquitylation activity.

## Discussion

Building on previous studies, the present study proposes and reinforces the need redefine of the HECT domain boundaries currently annotated on UniProt and the major databases. The HECT domain that was originally annotated using the BLAST search engine of the NCBI databases yielded only 14 hits [1]. The NCBI GenBank databases at the end of 1994 contained less than 238,000 total sequences as compared with nearly 250,000,000 sequences currently in the databases as of the writing of this study. The original boundaries for the HECT domain were defined from the sequences found in Pfam entry PF00643 and InterPro entry IPR000569. Subsequent HECT domain boundaries and analyses have used this definition as the basis for phylogenetic studies such as the identification of HECT proteins in soybeans [37].

Identifying the exact domain boundaries within a protein often requires access to solved structural models; however, bioinformatic approaches using MSA and secondary structure and disorder prediction have proved to be successful approaches to predict domain boundaries. For example, this bioinformatic MSA approach was used to successfully predict the boundaries for the novel Parkin RING0 domain, and the disordered regions found between domains such as the ubiquitin-like (UbLD) and RING-BRcat-Rcat (RBR) domains of Parkin [38] before 3D structures of full-length parkin were released in 2013 [39–42]. Although the PONDR results concerning the human HECT domains can show some differences, as it often predicts disorder in known structures at the N-terminal, the PONDR server reproducibly predicted that there was disorder before our new extended HECT domain boundaries. Previous bioinformatic analysis performed on the HECT family have identified conserved residues that regulate autoinhibition upon modification. As with NEDD4 and E6AP, there are lysine and threonine residues that reside within the first α-helix predicted to fold preceding the N-lobe that are subject to methylation and phosphorylation [43,44].

While it was found that this N-terminal extension also induces HECT-dependent oligomerization, the exact structural mechanisms used by many of the members in the HECT E3 ubiquitin ligase family that employ this modification remain only partially understood. Previous studies have also pointed out that the inclusion of the extended α-helices in-front of the HECT domain are necessary to perform structural analysis of the HECT domain that coincidentally also include highly conserved, essential residues that mediate HECT-dependent oligomerization [3,8,11,31,43,45]. Future studies are essential to examine the oligomerization and/or autoinhibition potential for the currently illusive members of the HECT E3 ubiquitin ligases.

Within the Human Gene Mutation Database (HGMD), numerous missense mutations have been annotated in the HECT E3 ubiquitin ligase family that are in proximity to the HECT domain (Table 1) [46]. Of these, some are relatively conserved and correlate to the onset of similar diseases and/or disorders. For example, an N-terminal missense mutation of an arginine to stop codon, irrespective of their subfamily classification, is linked to intellectual disability. Specifically assessing the HECW2 (R1330W) and HACE1 (R585W) mutants, this arginine residue is relatively conserved in the N-lobe and linked to the onset of neurological disorders [47,48]. Interestingly, the HECTD3 mutant (R478C), which has been linked to Tourette syndrome, lies i-3 from the conserved phenylalanine of the α1′-helical extension that protects the hydrophobic pocket. Also, the HECW2 F1193V missense mutation identified in neurodevelopmental delay and hypotonia is the conserved α1′ residue that interacts with the N-terminal α-helix to protect the hydrophobic pocket [49]. This demonstrates that some of these novel missense mutations within the HECT E3 ubiquitin ligase family likely are involved, whether directly or indirectly, with protection of this hydrophobic pocket to maintain its structure and ubiquitylation function.

**Table 1:** HECT domain mutations annoted in the HGMD database linked to disease

HECT	Residue substitution	Location in the HECT domain	Clinical manifestation
NED4L	Y679C; Q974H	N-lobe	Periventricular neuronal heterotopia [50]
	E893K; R897Q	C-lobe	
SMURF2	T641A	C-lobe	Neurodevelopmental disorder [51]
HECW2	R1191Q; F1193V	ext-α2^a^	Neurodevelopmental delay and hypotonia [49]
	R1330W	N-lobe	Developmental delay, absent speech, epilepsy, encephalopathy, hypotonia, and macrocephaly [47]
	D1442G		Autism spectrum disorder [52]
	E1445G		Neurodevelopmental delay and hypotonia [49]
HERC1	L4154*X*^b^	ext-α3^a^	Autism spectrum disorder [53]
	G4520E	N-lobe	Overgrowth, intellectual disability, and facial dysmorphism [54]
HERC2	D4267E	ext-α3^a^	Neurological disease [55]
TRIP12	R1595Q S1840L	N-lobe	Intellectual disability [56]
	Q1916*X*^b^	C-lobe	Intellectual disability [57]
HUWE1	R3267H	ext-α4^a^	Craniosynostosis [58]
	R4013W	ext-α1^a^	Mental retardation, X-linked [59]
	R4023C; N4075K; Y4106C; L4157V; E4244D		Intellectual disability [60]
	R4063Q	N-lobe	Intellectual disability, microcephaly, and postnatal growth failure [61]
	R4130Q		Developmental delay, Turner-type [62]
	R4187H		Intellectual disability, X-linked [63]
	R4187C		Mental retardation, X-linked [59]
	G4229D		Multiple congenital anomalies [64]
	K4295N	C-lobe	Intellectual disability [60]
	G4310R		Intellectual disability, microcephaly, and postnatal growth failure [61]
HACE1	R585W	N-lobe	Autism spectrum disorder [48]
	Gln618*fs*^c^; P674*fs*^c^; R748*X*^b^		Spastic paraplegia and psychomotor retardation with or without seizures [65,66]
	L832*X*^b^	C-lobe	
	A861P		Hereditary spastic paralegia [67]
HECTD3	R478C		Tourette syndrome [68]
AREL1	P779L	C-lobe	Pulmonary inflammation [69]
UBE3B	Q700*X;* G779R	N-lobe	Kaufman oculocerebrofacial syndrome [70]
	Q727P		Blepharophimosis-ptosis-intellectual-disability syndrome [71]
	R922C		Autism spectrum disorder [72]
	R997P	C-lobe	Kaufman oculocerebrofacial syndrome [70]
	Q1005P		Kaufman oculocerebrofacial syndrome [73]
UBE3C	S845F	N-lobe	Autism spectrum disorder [74]
	F996C	C-lobe	
E6AP	I827K; G870D	C-lobe	Angelman syndrome [75,76]

a ext-α = predicted α-helix N-terminal to current UniProt defined boundary

b *X* = stop codon

c *fs* = codon frameshift

HECT E3 ubiquitin ligases have been implicated in numerous diseases and disorders in relation to their altered expression and functional capabilities [2]. For example, the altered expression levels of HACE1 leads toward the development of Wilms’ tumor [77]. Interestingly, overexpression of HUWE1 has been correlated to breast, brain, and prostate cancer progression, but also is down-regulated in both colorectal and lung cancer [78]. The effect that these residue substitutions listed in Table 1 have on the structure and function of the HECT E3 ubiquitin ligase family members require further investigation to determine if these residue substitutions affect protein stability, autoregulation, or HECT domain concentration-dependent oligomerization.

While this manuscript was in preparation, the structure prediction AlphaFold database was publicly released [79]. The domain boundaries from our study and the predicted structures from AlphaFold are summarized in Table 2. The four largest human HECT E3 ubiquitin ligases are absent from the AlphaFold database as they exceed their 2700 amino acid length criterion (i.e. HERC1, HERC2, HECTD4, and UBR5). In general, there was consensus between the extended HECT and AlphaFold database domain boundary predictions with an obligate α-helix present to stabilize the HECT N-terminal lobe (Table 2).

**Table 2:** Uniprot PROSITE and predicted AlphaFold boundaries of the HECT E3 ligase family

Protein ID		UniProt	MSA/Jalview	AlphaFold	Notes
HERC Family	HERC1	4501-4848	4451-4861	N/A	-
	HERC2	4457-4794	4400-4834	N/A	-
	HERC3	951-1050	681-1050	659-1050	Longer predicted loop and helix
	HERC4	730-1057	689-1057	667-1057	Longer predicted loop and helix
	HERC5	702-1024	659-1024	638-1024	Longer predicted loop and helix
	HERC6	693-1017	650-1022	629-1022	Longer predicted loop and helix
NEDD Family	NEDD4	984-1318	940-1319	941-1319	PDB 2XBF; [33]
	NED4L	640-974	594-975	595-975	PDB 2ONI (X-ray: SGC)
	ITCH	569-903	527-903	528-903	PDB 3TUG (X-ray: SGC)
	WWP1	588-922	547-922	547-922	PDB 1ND7; X-ray [6]
	WWP2	536-870	494-870	494-870	PDB 4Y07; X-ray [32]
	SMURF1	420-757	377-757	369-757	Similar prediction
	SMURF2	414-748	371-748	367-748	PDB 1ZVD; X-ray [26]
	HECW1	1271-1606	1226-1606	1223-1606	Similar prediction
	HECW2	1237-1572	1192-1572	1189-1572	Similar prediction
Other Family	TRIP12	1885-1992	1561-1992	1558-1992	unique helix orientation PDB 3G1N, 3H1D, 5LP8;
	HUWE1	4038-4374	3993-4374	N/A	X-ray [27,31] PDB 7MWE; Cryo-EM [80]
	HACE1	574-909	529-909	524-909	Similar prediction
	HECTD1	2151-2610	2192-2610	2091-2610	Longer predicted loop and helix
	HECTD2	437-776	395-776	377-776	Longer predicted loop and helix
	HECTD3	512-857	474-861	451-861	Longer predicted loop and helix
	HECTD4	3627-3996	3570-3996	N/A	-
	UBR5	2462-2799	2395-2799	N/A	-
	AREL1	483-823	439-823	439-823	PDB 6JX5; X-ray [25]
	G2E3	371-698	341-706	345-706	Similar prediction
	UBE3B	702-1068	659-1068	650-1068	Longer predicted loop and helix
	UBE3C	744-1083	695-1083	695-1083	PDB 6K2C; X-ray [30]
	E6AP	776-875	500-875	486-875	Longer predicted loop and helix

**Figure 6:**
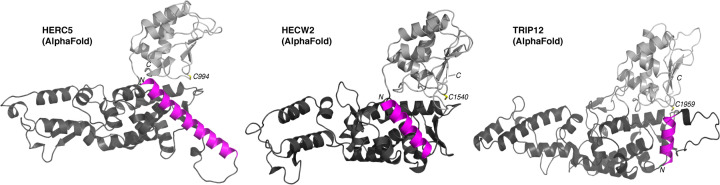
AlphaFold predicts an α-helix preceding the HECT domain Predicted models of the extended HECT domain of HECW2, HERC5, and TRIP12 from the AlphaFold database (https://alphafold.ebi.ac.uk/) showing different α-helical extension configurations. The HECT N-terminal lobe (dark gray), C-terminal lobe (light gray), α-helical extension that stabilizes the N-terminal lobe (magenta), and the catalytic cysteine (yellow) shown that AlphaFold models predicts that different orientations for the N-terminal extension in the extHECT construct may be possible.

The significant difference was the size of the loop between the canonical HECT and the helical extension, and this was most pronounced in the HERC subfamily (i.e. HERC1, HERC2, HERC3, HERC4, HERC5, and HERC6) as well as HECTD1, HECTD2, and HECTD3. It should also be noted that the extended α-helix is predicted to lie in the hydrophobic groove with its *N*-terminus proximal to the C-lobe, with the only exception being TRIP12 where the *N*-terminal helix is predicted to lie in the opposite orientation (Figure 6). Regardless, these new computational models further support our suggestion that the HECT domain boundaries need to be expanded to include the N-terminal helix.

In summary, our study demonstrates that our extHECT constructs have increased solubility, improved stability, higher yields, and robust ubiquitylation activity. While some HECT E3 ubiquitin ligases have solved HECT domain models [6,25–27,30,32,33], many others remain unknown. To date, most of these HECT domain structures have been solved for members of the NEDD4 subfamily, with only a few members in the ‘other’ subfamily having a HECT domain structure available on the Protein Data Bank. The present study clearly shows that extending the N-terminal lobe and redefining the HECT domain boundaries for all the human HECT E3 ubiquitin ligases is warranted. This knowledge will be an important consideration for future biochemical, structural, and therapeutic studies on the HECT E3 ubiquitin ligase family.

## Methods

### Bioinformatic analysis of the HECT E3 ubiquitin ligases

The sequences for the 28 human HECT paralogs were acquired from the UniProt database (www.uniprot.org) using the canonical HECT E3 ubiquitin ligase isoform and parsing out splice variants. The sequences were aligned using ClustalW [81] and T-Coffee [82] followed by manual curation in Jalview [28]. Secondary sequence prediction by JPred [83] was used on the aligned sequences within Jalview to assist in determining domain boundaries. Furthermore, the Protein Data Bank (PDB; www.rcsb.org) database was scoured to identify all the deposited PDB structures for isolated HECT domains for PyMol analysis [29,84] (Figures 1 and 2). Jalview was also used to assess the ortholog family of HACE1, showing both high conservation and α-helical prediction location immediately N-terminal to the annotated HECT domain (Figure 2B). Ortholog MSA analysis of both protein families were downloaded from Ensembl (ensembl.org) and analyzed in Jalview [28,85]. Sequences that were obvious fragments (lacking an intact HECT domain that included the catalytic cysteine), contained stretches of unknown residues, or isoforms from the same organism were removed from the analyses. The organisms were chosen to span disparate eukaryotic clades. PONDR VSL1 analysis was also used to search the HECT E3 ubiquitin ligases for predicted disordered regions to assist in domain boundary predictions (www.pondr.com). Expasy ProtParam was used to predict the physicochemical properties of the predicted extended HECT domain constructs (web.expasy.org/protparam) [86,87].

The proteins focused on in this study (HECW2, HERC4, G2E3, and HACE1) do not have any deposited PDB structures, and our initial studies with our constructs based upon UniProt HECT domain boundaries had proven difficult to express and purify for downstream biochemical and biophysical analysis. To determine the size of the hydrophobic patch, the PDB files for the known HECT structures that include the helical extension were analyzed using PyMol. The helical extension was removed from the original PDB file as the basis for the shorter HECT domain analysis. Only the A chain was considered, all waters and other heteroatoms removed, and hydrogens were added. Hydrophobic residues (A,G,V,I,L,F,M,Y,W) sidechain atoms only were selected and the PyMol command ‘get_area’ was used to quantitatively determine the surface area coverage.

### HECT domain expression plasmid design and construction

Expression plasmids containing the full-length open reading frame of the corresponding HECT E3 ubiquitin ligases were designed with *E. coli* codon optimization and synthesized by Synbio Technologies, Inc. (Monmouth Junction NJ, U.S.A.). The plasmids contained the essential components desired for protein overexpression in *E. coli* – a T7 promoter induced through the addition of ß-D-1-thiogalactopyranoside (IPTG), a lac operon, origin of replication and terminator sequence, antibiotic resistant marker for ampicillin (amp) for selection, an N-terminal His_6_-SUMO tag for increased protein solubility and downstream Ni^2+^-immobilized metal affinity chromatography (Ni^2+^-IMAC) purification, and flanking restriction sites for *Bam*HI (5′) and *Xho*I (3′) in the multiple cloning site.

Primers were designed to isolate both the UniProt defined HECT (HECT) and extended HECT (extHECT) domains for each protein from the full-length sequence plasmids by deleting residues from the full-length vector using single primers in parallel (SPRINP) [88] and quick-change mutagenesis (QCM) [89] strategies (Table 3). The boundaries for each extHECT construct were chosen based on bioinformatic predictions including the MSA and reference to the recent AREL1 and UBE3B HECT domain PDB structures [25,30]. These included the NEDD4 family member HECW2 (HECT: 1237-1572; extHECT: 1190-1572), HERC protein HERC4 (HECT: 708-1057; extHECT: 675-1057), and ‘other’ HECT subfamily members G2E3 (HECT: 369-706; extHECT: 340-706) and HACE1 (HECT: 574-909; extHECT: 523-909). All primer sequences were synthesized by Thermo-Fisher. SPRINP and QCM were performed using a T100 Thermal Cycler (Bio-Rad). The HECW2 and HERC4 extHECT expression constructs were made through PCR amplification of the desired ORF, restriction digestion with flanking *Bam*HI (5′) and *Xho*I (3′), and ligation with T4 DNA ligase into the His_6_-SUMO plasmid. Colonies were screened by colony PCR and restriction digestion, and positive transformants were verified by Sanger DNA sequencing (Psomagen, Cambridge MA, U.S.A.).

**Table 3:** Oligonucleotide primers for recombinant HECT and extHECT plasmids

Primers	Orientation	Nucleotide sequence
HACE1		
572-909	Sense	5′-GGAGGTGGATCCAAGGCTAATTGTGCTAAG-3′
	Antisense	3′-CTTAGCACAATTAGCCTTGGATCCACCTCC-5′
523-909	Sense	5′-GGAGGTGGATCCCAGCCGTTCAAGGATCGC-3′
	Antisense	3′-GCGATCCTTGAACGGCTGGGATCCACCTCC-5′
G2E3		
369-706	Sense	5′-GATTGGAGGTGGATCCACGAAGCGTCTTTAC-3′
	Antisense	3′-CTAACCTCCACCTAGGTGCTTCGCAGAAATG-5′
340-706	Sense	5′-GATTGGAGGTGGATCCTCAAAATTCCGGCG-3′
	Antisense	3′-CTAACCTCCACCTAGGAGTTTTAAGGCCGC-5′
HECW2		
1237-1572	Sense	5′-GGAGGTGGATCCAGCAGGAAGGAC-3′
	Antisense	3′-CCTCCACCTAGGTCGTCCTTCCTG-5′
1190-1572	Sense	5′-GGAGGTGGATCCAAACGCGATTTCG-3′
	Antisense	3′-CCTCCACCTAGGTTTGCGCTAAAGC-5′
HERC4		
708-1057	Sense	5′-CAGATTGGAGGTGGATCCTGCCTAATCTTGGTT-3′
	Antisense	3′-GTCTAACCTCCACCTAGGACGGATTAGAACCAA-5′
683-1057	Sense	5′-CAGATTGGAGGTGGATCCCAGATGGCGATTGAC-3′
	Antisense	3′- GTCTAACCTCCACCTAGGGTCTACCGCTAACTG-5′

### Overexpression and purification of HECT and extHECT domains

The His_6_SUMO-HECT and His_6_SUMO-extHECT expression plasmids for HECW2, HERC4, G2E3, and HACE1 were transformed into either BL21(DE3) or LOBSTR(DE3) *E. coli* cell lines and grown at 37°C at 200 rpm in Luria-Burtani media supplemented with 100 μg/ml ampicillin. When the cultures reached an OD_600_ of approximately 0.6–0.8, the cultures were induced with 0.5 mM IPTG and incubated at 16°C at 200 rpm for 16–20 h. The cells were harvested by centrifugation at 10,000 rpm for 10 min at 4°C using a Sorvall LYNX 4000 superspeed centrifuge with a Fiberlite F10-4x1000 LEX Carbon Fiber rotor (Thermo-Fisher). Cell pellets were resuspended with either a Tris or phosphate-based Wash Buffer (50 mM Na_2_HPO_4_ or Tris-HCl, 300 mM NaCl, 10 mM imidazole, pH 8.0) supplemented with ProBlock™ Protease Inhibitor Cocktail (GoldBio, St. Louis MO, U.S.A.; 100 μl/ml), lysed using an EmulsiFlex-C5 homogenizer (Avestin, Ottawa ON, Canada), and clarified by ultracentrifugation using a Optima L-80 XP ultracentrifuge with a 70.1 Ti rotor (Beckman-Coulter) at 41,000 rpm for 40 min. The supernatants were then passed through a 0.45 μm syringe sterile filter (Thermo-Fisher) prior to being applied to 10 ml of Ni^2+^-IMAC resin (GoldBio) pre-equilibrated with wash buffer. After the resin was extensively washed with 25 column volumes of wash buffer, the HECT or extHECT protein was eluted with 30–50 ml of Elution Buffer (50 mM Na_2_HPO_4_ or Tris-HCl, 300 mM NaCl, 250 mM imidazole, pH 8.0). Fractions containing eluted protein were pooled and incubated with recombinant SUMO protease (Ulp1; 40 μg/ml) to cleave the His_6_SUMO tag, then dialysed against Wash Buffer in 3.5 kDa MWCO Spectra/Por 3 Dialysis Tubing (Thermo-Fisher) at 4°C with stirring overnight. The Ulp1 cleaved protein was then reapplied to 10 ml of equilibrated Ni^2+^-IMAC resin in Wash Buffer and the flowthrough containing the desired HECT or extHECT protein was collected and pooled. The protein was then concentrated using an Amicon 15 mL centrifugal filter with a 10 kDa MWCO (Millipore) and loaded on to a HiLoad 16/60 Superdex 75 or 200 column (Cytiva Life Sciences) equilibrated with Gel Filtration Buffer (20 mM Tris-HCl, 100 mM NaCl, 1 mM TCEP, 1 mM EDTA) at a flow rate of 1 ml/min using an ÄKTA Pure 25L FPLC or ÄKTA Start FPLC system (Cytiva Life Sciences). Fractions containing pure HECT or extHECT protein, as assessed by 15% SDS-PAGE and visualized with an iBright FL1000 gel imager (Thermo-Fisher), were pooled, aliquoted, flash frozen in N_2(l)_, and stored at −80°C.

### *In vitro* fluorescent ubiquitylation assay

All soluble HECT and extHECT proteins were assessed for their ability to catalyze ubiquitin transfer between all cascading enzymes using a fluorescent auto-ubiquitylation assay. Specific attention was given to observing if the inclusion of the N-terminal extension impacted HECT-dependent ubiquitylation activity. Once HECT/extHECT∼ubiquitin charging was confirmed through assay optimization, ubiquitylation assays were conducted with 5 μM Alexa Fluor 647 N-terminally tagged ubiquitin (0.1 μM E1 activating enzyme UBE1 (Uba1), 10 μM E2 conjugating enzyme UBE2L3 (UbcH7) or UBE2D3 (UbcH5c), and 10 μM HECT or extHECT, 0.5 mM DTT, 4 μM ATP, 10 μM MgCl_2_ in 50 mM HEPES pH 7.5, 100 mM NaCl. Each reaction was incubated at 37 °C at 200 rpm for 1 to 2 h. To determine the presence of ubiquitin∼thioester intermediates, appropriate samples were supplemented with 4 mM DTT. Reactions were terminated by adding SDS-PAGE loading dye and heating at 95 °C in a dry bath for one minute. The samples were then loaded on to a 12% Bis-Tris gel at pH 6.4 and run for 1 h at 130 V. The gels were removed from the apparatus and immediately visualized on an iBright FL1000 imaging system (Thermo-Fisher) using the setting for Alexa Fluor 647 fluorescence gel imaging.

## Data Availability

All materials for this project could be made available upon request.

## Abbreviations

AREL1: apoptosis-resistant E3 ubiquitin protein ligase-1
E6AP: E6-associated protein
HECT: homologous to E6AP C-terminus
HGMD: Human Gene Mutation Database
MSA: multiple sequence alignment
RLD: RCC1-like domain
